# Erythrodermic psoriasis treatment with Guselkumab: report of two cases and literature review^☆^

**DOI:** 10.1016/j.abd.2023.05.007

**Published:** 2023-12-07

**Authors:** Esperanza Welsh, Jesus Alberto Cardenas-de la Garza, José Alberto García-Lozano, Diana Paola Flores-Gutierrez

**Affiliations:** a Welsh Dermatology and Associates, Monterrey Nuevo Leon, Mexico; b Rheumatology Departament, Universidad Autónoma de Nuevo León, Hospital Universitario ‘Dr José Eleuterio González’, Monterrey NL, México; c Department of Clinical Introduction, Faculty of Medicine and University Hospital “Dr. José E. González”, Autonomous Universitiy of Nuevo León, Monterrey, Nuevo León, Mexico

Dear Editor,

Erythrodermic psoriasis (EP) is an uncommon and possibly fatal psoriasis presentation involving more than 80% of the body surface area (BSA).^1^ Controlled clinical trials and current treatment choices for EP are limited and, compared to plaque-type psoriasis, EP patients seem to have a worse clinical response to standard therapies.^1^ Guselkumab is a human monoclonal antibody against Interleukin-23 (IL-23), that joins the p19 subunit of IL-23 and has exhibited excellent and sustained treatment effects in moderate-to-severe plaque-type psoriasis.^1^ However, reports of guselkumab efficacy in EP are scarce. This study aims to report two cases of EP treated with guselkumab with sustained efficacy and perform a literature review of guselkumab in the treatment of EP.

We present a 65-year-old woman with a 4-year history of psoriasis previously treated with topical steroids. Her disease flared involving >90% of her BSA. Skin examination demonstrated symmetrical erythematous scaly plaques (Fig. 1A). A punch biopsy was compatible with psoriasis. Guselkumab (100 mg via subcutaneous injection at week 0 and week 4, followed by a dose every 8 weeks), topical steroids, and emollients were started. The patient achieved a complete response (PASI100) by week 12 (Fig. 1B) and has maintained it for over 32 months. The second case is a 51-year-old man with a 1-year history of plaque-type psoriasis treated with topical steroids. He presented with erythematous plaques on his extremities and trunk that spread to a PASI 40. Histopathological analysis was compatible with psoriasis. Treatment with guselkumab as mentioned previously resulted in complete resolution by week 12 which has persisted for 2-years.

**Figure 1:**
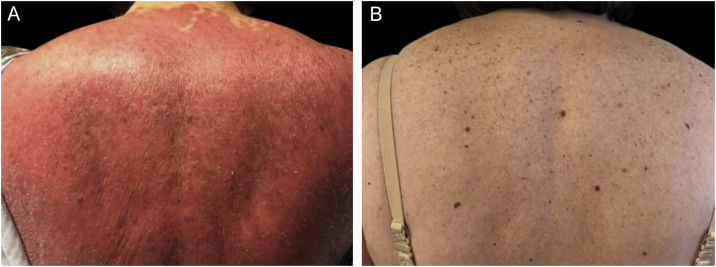
(A) Erythrodermic psoriasis; (B) Remission after guselkumab therapy.

We performed a literature review of EP treated with guselkumab on August 15, 2022, through MEDLINE (PubMed) with keywords erythroderm* AND guselkumab. Of the 10 results, we excluded 3 as they were about other conditions (non-erythrodermic psoriasis, pustulotic arthro-osteitis, palmoplantar pustulosis, palmoplantar psoriasis, psoriatic arthritis, erythrodermic ichthyosis) and 3 that were reviews.

We included 4 articles with 26 patients combined with EP in treatment with guselkumab. Most patients were men (n = 24), and the mean age was 49.9 years.^1^, ^2^, ^3^, ^4^ All patients showed a good response during treatment, except one with concomitant Castleman’s disease and one that withdrew consent from the study.^1^, ^2^, ^3^, ^4^ Sano et al.^3^ reported 10 (90.9%) patients with “treatment success” at week 16. Ten (90.9%) patients reported a mean PASI of 3.9 (SD = 4.27) with a median improvement of 94.1% by week 52. Chiang et al.^1^ reported 13 patients in follow-up for 28 weeks, where 8 (61.5%) reached PASI 50 response by week 12. Megna et al.^2^ reported one patient with PASI 100 at 20 weeks and sustained effect by week 48. Zanelli et al.^4^ reported a patient with multicentric Castleman’s disease and EP that did not respond to guselkumab therapy.

The two patients reported herein had a PASI 100 response by week 12 with sustained effect at the last follow-up at 24 and 32 months and no adverse events. IL-23 inhibitors have shown higher PASI90 and PASI100 response rates compared to anti-TNF alpha inhibitors in moderate and severe psoriasis with a similar adverse event profile.^5^ Our review found few cases and case series of EP treated with guselkumab but a high response rate. Reported adverse events were infrequent and mild. Several factors influence treatment decisions, including infections (e.g., tuberculosis or hepatitis B/C), affordability, comorbidities, and accessibility. Our study suggests that guselkumab is an efficient treatment for EP, given the results, safety, and long-term effectiveness it has shown. Comparative studies, that include other biologics like risankizumab and tildrakizumab, are needed to define the best treatment for patients with EP.

## Financial support

None declared.

## Authors’ contributions

Esperanza Welsh: Collected the clinical data and reviewed the draft of the manuscript. Approval of the final version of the manuscript.

Jesus Alberto Cardenas-de la Garza: Collected the clinical data, adapted the clinical image, and wrote a draft of the manuscript. Approval of the final version of the manuscript.

José Alberto García-Lozano: Collected the clinical data and obtained the figure. Approval of the final version of the manuscript.

Diana Paola Flores-Gutierrez: Collected the clinical data and wrote a draft of the manuscript. Approval of the final version of the manuscript.

## Conflicts of interest

Esperanza Welsh has been a consultant and/or speaker for Merz, Leo Pharma, and Janssen.

Jesus Alberto Cardenas-de la Garza has been a consultant for Leo Pharma.

José Alberto García-Lozano and Diana Paola Flores-Gutierrez have nothing to disclose.
